# Meta-analysis of the efficacy of preoperative skin preparation with alcoholic chlorhexidine compared to povidone iodine in orthopedic surgery

**DOI:** 10.1038/s41598-021-97838-8

**Published:** 2021-09-20

**Authors:** Mario Mastrocola, Georg Matziolis, Sabrina Böhle, Chris Lindemann, Peter Schlattmann, Henk Eijer

**Affiliations:** 1 Department of Orthopaedic Surgery, Spital Emmental, Oberburgstrasse 54, 3400 Burgdorf, Switzerland; 2 Orthopaedic Department, Campus Eisenberg, Jena University Hospital, Klosterlausnitzer Str. 81, 07607 Eisenberg, Germany; 3 Department of Medical Statistics, Computer Sciences and Documentation, Jena University Hospital, Bachstr. 18, 07743 Jena, Germany

**Keywords:** Medical research, Risk factors

## Abstract

Preoperative skin preparation is an effective method to prevent surgical site infections (SSI). Alcoholic chlorhexidine (CHG) and povidone iodine (PV-I) are the most widely used antiseptic agents. This meta-analysis aims to determine their efficacy in reducing natural bacterial skin flora in clean orthopedic surgery. A systematic search was conducted through current literature up to June 2021 to identify clinical randomized trials that compared the efficacy of alcoholic chlorhexidine and povidone iodine in reducing bacterial skin colonization after preoperative skin preparation. A meta-analysis was conducted. Of 235 screened articles, 8 randomized controlled trials were included. The results of the meta-analysis demonstrate a significantly lower positive culture rate in the chlorhexidine group than in the povidone iodine group (RR = 0.53, 95% Cl: 0.32–0.88). The present data show the superiority of chlorhexidine in reducing the normal bacterial flora compared to povidone iodine in clean orthopedic surgery.

## Introduction

Despite a low rate of surgical site infections (SSI) in clean orthopedic interventions, varying between 0.3% and 1.9%^1–3^, their consequences, especially deep infections, are severe for the generally healthy patient. Therefore, management to reduce such complications remains important. The main source of SSI is the patient’s own normal skin flora and not contamination by instruments or room air. Topical antiseptic preparation of the skin plays a central role in reducing the burden of normal skin flora^4–7^. Because of its histologic structure, with glands and hair follicles in deeper skin layers, the human skin cannot be sterilized even by intense disinfection^8^. Therefore, the goal of surgical skin preparation remains to reduce bacterial colonization. As no antiseptic agent can sterilize tissue, the reduction of bacterial colonization depends on concentration and exposure to the antiseptic agent^7,9^. The concentration and exposure time have been established as a compromise between tissue toxicity and practicability of the different antiseptic agents in order to achieve a minimum of tissue toxicity and a short exposure time with a maximum reduction of the normal skin flora.

Most available data on preoperative skin preparation are only of moderate quality and are characterized by trials that are underpowered to detect rather rare events such as SSI^2,10,11^. The measurement of positive skin culture is a method used to compare the efficacy of antiseptic preparations. On the basis of this method, the efficacy of the two most widely used antiseptic agents in reducing bacterial colonization was determined according to the current literature.

By screening and analyzing the current orthopedic literature, the objective of this study was to determine the efficacy of preoperative skin preparation with alcoholic chlorhexidine compared to povidone iodine in reducing the natural bacterial skin flora.

## Methods

### Search strategy

The literature search was conducted systematically based on PubMed, Cochrane, Embase, Google Scholar, Web of Science, CINAHL, SPORTDiscus, and Scopus databases. The following search terms (MeSH terms) were used in combination: “chlorhexidine” AND “shoulder”, “elbow”, “hip”, “knee” “spine”, “ankle”, or “foot”, respectively. Original articles published in English up to June 2021 were included. References of retrieved articles were also screened for any potential studies.

### Inclusion and exclusion criteria

Studies were considered eligible if they met all of the following criteria:

1. published clinical randomized controlled trial (Level I study)
2. the experimental group received alcoholic chlorhexidine solution for preoperative skin preparation and the control group received a standard preoperative skin preparation with povidone iodine solution without alcohol and
3. the outcome was the measurement of bacterial colonization as a positive bacterial culture directly after skin preparation.

In vitro and animal studies were excluded. Studies focusing on pre-admission home treatment or clinical outcome data were also excluded.

### Selection process

To assess eligibility and review potential, a stepwise procedure was performed. First the recorded titles of the literature search were screened by applying the inclusion criteria, followed by analyzing the abstracts of the selected studies. Second, the remaining articles were assessed for eligibility by full text review.

### Data extraction

The data extracted for each study was the number of positive bacterial colonies after surgical skin preparation (post-preparation) with either chlorhexidine or povidone iodine solution. In all studies, culture swabs were taken systematically as samples after skin preparation, partially from multiple locations, and sent to a microbiology laboratory for analysis. Additionally, the number of postoperative surgical site infections was extracted for analysis. Other data on the management to reduce bacterial colonization could only be analyzed qualitatively, because the methods were not consistent.

### Data analysis and statistical methods

Meta-analysis was carried out using RStudio (version 0.97.551, RStudio Inc.). A fixed effect model was utilized. Additionally, the data were pooled and a Chi-square test was performed. For all statistical tests the level of significance was set to 0.05.

## Results

**Figure 1:**
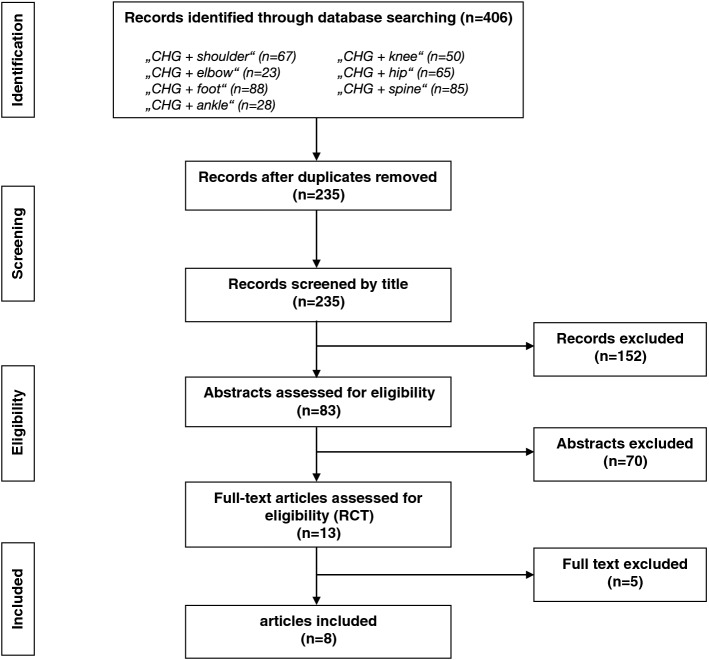
Flow diagram of literature search and study selection. *CHG* chlorhexidine, *RCT* randomized trial.

After removing duplicates, a total of 235 titles were screened and 83 abstracts assessed for eligibility. Of these, 70 abstracts were excluded based on the exclusion criteria and 13 randomized clinical trials remained for full text analysis. In this last step, 5 randomized controlled studies were excluded, because they examined the contamination of suture material or compared surgical skin preparation with chlorhexidine with an antiseptic solution other than povidone iodine^12–15^. Eight of the 13 full text analyzed articles^16–23^ compared a surgical skin preparation with alcoholic chlorhexidine vs. povidone iodine and were included in the meta-analysis (Fig. 1).

A total of 600 subjects were included in the 8 studies, which were published between 2005 and 2021 (Table 1). Subjects were aged from 17–88 years and gender was equally distributed. All studies excluded patients with open wounds, current infections, antimicrobial therapy or immunosuppression. In six studies, all patients had a surgical intervention with administration of preoperative antibiotics. All the surgical interventions were elective clean orthopedic surgeries, with the exception of one study, in which the patients had proximal humeral fracture surgery and underwent an open reduction and osteosynthesis^17^. Two studies were non-surgical trials, in which the patients were not operated on but had surgical skin preparation in an interventional setting as if an operation would follow^22,23^. Three studies were performed on shoulders, one on the spine and four on foot and ankle surgery. No adverse effects of the antiseptic substances were reported.

**Table 1:** Summary of randomized controlled studies included, comparing chlorhexidine with povidone iodine for preoperative skin preparation to reduce bacterial contamination.

Author	Year	Age	Gender m/f	Patients	Samples CHG/PV-I	Type of surgery	Surgical skin preparation CHG group	Surgical skin preparation PV-I group	PV-I with alcohol	Overall positive culture pre-prep %	Positive culture pre-prep % (n) CHG/PV-I	SSI CHG/PV-I
Blonna D. et al	2018	45–88	8/32	40	40/40^a^	Shoulder	Chlorohexidine gluconate 4% + povidone iodine 1% (10% iodine and 50% isopropyl alcohol)^b^	Povidone iodine 1% (10% iodine and 50% isopropyl alcohol)	Yes	92.5%	27.5% (11)/77.5% (31)	No
Saltzman MD. et al	2009	17–79	59/41	100	50/50	Shoulder	ChloraPrep (2% chlorhexidine gluconate and 70% isopropyl alcohol)	Povidone iodine (0.75% iodine scrub and 1% iodine paint)	No	85%	7% (4)/31% (16)	No
Yoshii T. et al	2018	51–79	89/101	190	98/92	Spine	0.5% chlorhexidine gluconate with 79% ethanol	10% PV-I (chemical complex of polyvinylpyrrolidone and elemental iodine; isodine)	No	83.7%	3.1% (3)/5.1% (5)	1/3 not significant
Shadid MB. et al	2019	22–75	7/42	49	26/23	Foot and ankle	0.5% chlorhexidine/70% alcohol	1% iodine/70% alcohol	Yes	69.4%	1.9% (1)/6.5% (3)	1/2 not significant
Cheng K. et al	2009	43–68	12/38	50	75/75	Foot and ankle	Alcoholic chlorhexidine (clear chlorhexidine gluconate 0.5% w/v in 70% v/v)	Alcoholic betadine (povidine iodine 10% w/w (1% w/w available iodine)	Yes	82.6%	6% (5)/12% (9)	No
Bibbo C. et al	2005	16–85	61/66	127	60/67	Foot and ankle	Chlorhexidine gluconate 4% scrub (7 min) and isopropyl alcohol 70% (paint)	Povidone iodine 7.5% scrub (7 min) and povidone iodine 10% solution (paint)	Yes	Not determined	38% (23)/79% (53)	No
Becerro de Bengoa Vallejo R. et al	2009	18–80	16/12	28	28/28^a^	Foot and ankle(no surgery)	4% chlorhexidine gluconate scrub for 5 min + 70% isopropyl alcohol paint	Prewash with 70% isopropyl alcohol for 3 min + 7.5% povidone iodine scrub for 5 min + 10% povidone iodine paint	Yes	62.5%	0/0	Non-surgical study
Doerfel et al	2021	22–74	7/6	16	16/16^a^	Shoulder (no surgery)	ChloraPrep (CHG 2% with 70% isopropyl alcohol)	Betaseptic (3.24% povidone iodine with 37.3% ethanol	Yes	100%	62.5% (10)/25% (4)	Non-surgical study

^a^Contralateral side used as controls, ^b^double skin preparation. CHG chlorhexidine, *PV-I* povidone iodine, *pre-prep* pre-preparation, *SSI* surgical site infection.

**Figure 2:**
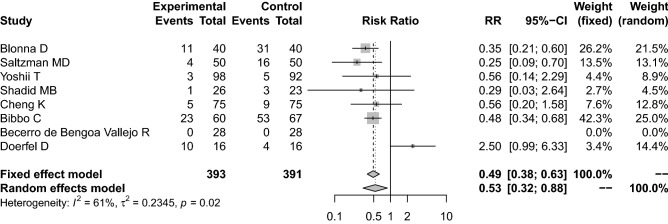
Meta-analysis comparing preoperative skin preparation with chlorhexidine versus povidone iodine in the reduction of positive bacterial cultures. *CHG* chlorhexidine, *PV-I* povidone iodine, *pos. BC* positive bacterial culture, *RR* risk ratio, *CI* confidence interval.

All studies reported a significant and effective reduction of the normal bacterial skin flora by surgical skin preparation with both solutions used. Four of the eight randomized controlled trials demonstrated no significant difference in the reduction of positive culture rate between the chlorhexidine and povidone iodine group. The meta-analysis of all eight studies showed significant heterogeneity among the studies (I^2^ = 61%, τ^2^ = 0.2345, *p* = 0.02) and therefore a random effect model was used. The pooled results demonstrated that the positive culture rate post-preparation was significantly lower in the chlorhexidine group than in the povidone iodine group (RR = 0.53, 95% CI: 0.32 to 0.88, *p* < 0.014; see Fig. 2). In addition, the pooled chi square test was significant (*p* < 0.001).

Although SSI rate was not the primary outcome parameter, most studies included this important clinical outcome measure. SSI occurred in only 2 studies^20,21^, 2 in the CHG and 5 in the PV-I group. In a further 4 studies no postoperative infection occurred^16–19^, while two were non-surgical studies^22,23^. These two were excluded and the meta-analysis showed no significant heterogeneity among the studies (I^2^ = 0%, τ^2^ = 0, *p* = 0.83) and therefore a fixed effect model was used.

**Figure 3:**
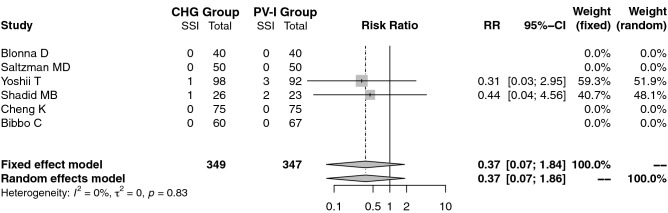
Meta-analysis comparing preoperative skin preparation with chlorhexidine versus povidone iodine in the prevalence of postoperative surgical site infections (surgical studies). *SSI* surgical site infection, *CHG* chlorhexidine, *PV-I* povidone iodine, *RR* risk ratio, *CI* confidence interval.

As a result of the small number of SSI events detected, the pooled results did not show any significant difference in the postoperative infection rate between the chlorhexidine group and the povidone iodine group (RR = 0.37, 95% CI: 0.07 to 1.86, *p* > 1) (Fig. 3).

## Discussion

The main result of this study is that surgical skin preparation with chlorhexidine alcohol is more effective in reducing the number of bacteria on human skin than povidone iodine.

Regarding the prevention of SSI, there is evidence in favor of CHG compared with PV-I, even if the available studies are heterogeneous (different kind of surgery, formulations, methods of application, duration, endpoints) and of moderate quality^2,24^. The evidence level as well as the numbers of included patients of most studies are low. Concentrations of chlorhexidine vary between studies (0.5% to 4%). Additionally, the mode of application of the disinfectants is not given in all or varies between studies. These points limit data comparability and any conclusions drawn from pooled data in this meta-analysis.

A meta-analysis by Zhang et al. on the prevalence of SSI in clean and clean-contaminated surgery demonstrated that preoperative skin preparation with CHG, compared with PVI, was associated with a lower incidence of SSI^25^.

In this study, there is no significant difference in the incidence of SSI between the two groups. The significance of this finding is low because of a low power and SSI was not the main focus. Moreover, the reduction of bacterial load on the skin cannot be translated directly into a reduction of SSI, as it is a multiple factor complication. Beside the reduction of bacterial load, local and systemic immunity, previous medical optimization (anemia, albuminemia, glycemia control, etc.) and perioperative maintenance of normothermia, and optimal oxygenation have an impact on the prevalence of SSI^6,26^.

Staphylococcus aureus (20%) and coagulase-negative staphylococci (CoNS) (14%) are the most frequent causes of surgical site infections in surgery overall^4,27,28^. CoNS are most abundant in normal bacterial flora^21,29^, and are also the most commonly isolated species after preoperative skin preparation in the other studies included^17–20,22,23^, as described previously^30^. The rate of positive cultures for CoNS can be explained by its normal abundance and a high bacterial load before surgical skin preparation.

Regarding Cutibacterium acnes, which is a major pathogen causing deep SSI in shoulder surgery^31,32^, neither surgical skin preparation significantly reduced the positive culture rate post-preparation^17,19^. This is consistent with other studies, which found a frequent isolation of Cutibacterium acnes after surgical skin preparation in shoulder surgery on skin and dermal biopsies^30,33–35^. The persistent colonization of Cutibacterium acnes may be due to its association with sebaceous glands and deeper skin layers, making it difficult to assess after a topical disinfectant^12,31,36^.

With the exception of Becerro et al., residual bacterial species were found after surgical skin preparation in all of the studies presented. Residual bacterial species after skin preparation lead to recolonization during surgery. Yoshii et al. found a higher positive culture rate after wound closure of 8.4% compared to 4.2% directly after surgical skin preparation. This finding is supported by several other authors and indicates that there is a kind of bacterial reservoir that is insufficiently addressed by topical skin preparation methods^13,21,23,37,38^.

These findings suggest that not only should surgical skin preparation be optimized to minimize the normal skin flora but that other strategies must also be considered, such as reducing surgery duration, and adjusting the timing and type of systemic antibiotic prophylaxis, in order to reduce the rate of surgical site infections to an absolute minimum^6,33,37^.

Although both antiseptic solutions possess a similar spectrum of antimicrobial activity, they differ in their mechanisms, rapidity and persistence of action^4,39^. Chlorhexidine is a chlorinated biguanide that directly disrupts the cell membrane within a wide pH range (5–8), whereas povidone iodine affects the structure and function of proteins by oxidation via free iodine at a narrow pH (around 6)^18,20^. Studies evaluating the pharmacokinetics have shown an antimicrobial activity of iodine for 3 hours^40^, whereas chlorhexidine has a residual effect for 6 hours^41^. One disadvantage of povidone iodine is that it can be inactivated by protein material^39^. In contrast, chlorhexidine remains active despite bodily fluids and is therefore successfully used in oral surgery^42^. This may explain why chlorhexidine remains active for longer than povidone iodine.

Compared to povidone iodine, which is mostly used in aqueous solutions, chlorhexidine is dissolved in alcoholic solutions in concentrations ranging from 70 to 90%.

These alcoholic solutions exhibit a very rapid broad-spectrum antimicrobial activity by denaturing proteins, also against mycobacteria and fungi, against which chlorhexidine has a lower activity^4,7,43^. Efficacy is highest at concentrations between 70 and 90%, but they lack residual activity because of their volatility^4^.

Alcoholic chlorhexidine solutions therefore present a combined effect of both substances. However, a study conducted on healthy subjects comparing the efficacy of aqueous chlorhexidine with that of alcoholic chlorhexidine could not find a significant difference^44^.

Becerro et al. showed a similar synergistic effect of povidone iodine in alcoholic solution. They report a significant efficacy in reducing the positive culture rate overall and especially for CoNS using povidone iodine with alcohol, compared to povidone iodine alone. However, a Cochrane review found no difference in efficacy between aqueous and alcoholic solutions^2^.

A Cochrane review and meta-analysis on the effectiveness of preoperative antisepsis in preventing SSI found no statistically significant difference in the number of SSI following skin preparation with alcoholic or aqueous solutions in the studies comparing them directly, even though the data are in favor of alcoholic solutions. The mixed treatment comparison suggests that alcoholic chlorhexidine is most effective in preventing SSI, but the low quality of evidence is pointed out^2^.

In the present study, alcoholic chlorhexidine solutions were even more effective in reducing skin bacteria than povidone iodine with alcohol. It can be concluded that alcohol has a synergistic effect with both povidone iodine and chlorhexidine and that the latter most effectively reduces skin bacteria due to its antimicrobial activity, rapidity and persistence of action.

Alcoholic disinfectants can act as fuel in surgical fires, causing severe skin burns. These surgical fires are rare and considered as one of the few “never events”. Such events can be prevented by allowing the disinfectants enough time to dry, avoiding liquid pooling, and using soaked drapes^45–47^.

The methods of application (i.e. duration, scrub or paint, etc.) used in the studies included here are very heterogeneous and their impact on bacterial reduction remains unclear. Recommendations by the AAOS or MSIS are lacking and therefore manufacturers’ guidelines should be followed.

In conclusion, chlorhexidine and povidone iodine, the solutions most commonly used in preoperative skin antisepsis, are both effective in reducing SSI. The present data additionally demonstrate the superiority of alcoholic chlorhexidine in reducing the normal bacterial flora compared to povidone iodine, especially in clean orthopedic surgery.

Nonetheless high quality studies are needed to address prospective randomized and blinded clinical trials to draw definite clinical conclusions.
